# Effect of Er on Microstructure and Corrosion Behavior of Al–Zn–Mg–Cu–Sc–Zr Aluminum Alloys

**DOI:** 10.3390/ma15031040

**Published:** 2022-01-28

**Authors:** Qingyuan Xing, Xiaohui Wu, Jinxin Zang, Linggang Meng, Xingguo Zhang

**Affiliations:** 1 AECC Beijing Institute of Aeronautical Materials, Beijing 100095, China; apple_zjx@163.com; 2 School of Materials Science and Engineering, Dalian University of Technology, Dalian 116024, China; 18235162487@163.com (X.W.); zxgwj@dlut.edu.cn (X.Z.)

**Keywords:** Al–Zn–Mg–Cu–Sc–Zr alloy, evolution of Er phase, intergranular corrosion

## Abstract

In this study, the influence of Er addition on the microstructure, type transformation of second phases, and corrosion resistance of an Al–Zn–Mg–Cu alloy were explored. The results revealed that the added Er element could significantly refine the alloy grains and change the second-phase composition at the grain boundary of the alloy. In the as-cast state, the Er element significantly enhanced the corrosion resistance of the alloy due to its refining effect on the grains and second phases at the grain boundary. The addition of the alloying element Er to the investigated alloy changed the type of corrosion attack on the alloy’s surface. In the presence of Er, the dominant type of corrosion attack is pitting corrosion, while the alloy without Er is prone to intergranular corrosion attack. After a solution treatment, the Al_8_Cu_4_Er phase was formed, in which the interaction with the Cu element and the competitive growth relation to the Al_3_Er phase were the key factors influencing the corrosion resistance of the alloy. The anodic corrosion mechanism of the Al_8_Cu_4_Er and Al_3_Er phases evidently lowered the alloy corrosion rate, and the depth of the corrosion pit declined from 197 μm to 155 μm; however, further improvement of corrosion resistance was restricted by the morphology and size of the Al_8_Cu_4_Er phase after its formation and growth; therefore, adjusting the matching design of the Cu and Er elements can allow Er to improve the corrosion resistance of the Al–Zn–Mg–Cu aluminum alloy to the greatest extent.

## 1. Introduction

In the aerospace field, the 7xxx series aluminum alloy has been widely applied due to its high strength, high modulus, and low density; however, its corrosion resistance is not ideal, which seriously restricts the safe use of this alloy series [1,2,3]. Adding a trace amount of rare earth and transition elements can markedly refine the grains, improve the dendritic segregation, and enhance the corrosion resistance of the alloy [4,5,6,7]. Sc can achieve a more obvious effect because, once added, it contributes to the formation of primary and secondary Al_3_Sc phases. These phases not only serve as the core of heterogeneous nucleation to refine the grains and improve the segregation, but also effectively impede the migration of dislocations and sub-boundaries and strengthen the comprehensive mechanical properties of the alloy [8,9,10]; however, Sc cannot be extensively applied to industrial production because of its very high cost. Al_3_Er is generated by Erbium, which is cheaper than Sc. In an aluminum alloy, it is an LI_2_-type compound similar to Al_3_Sc, belongs to the Pm3m space group (AuCu_3_ crystal structure), has a good coherent relationship with the matrix, and can be used as an effective strengthening phase for the alloy. Moreover, it can effectively stabilize the substructures and repress the recrystallization and is therefore regarded as an ideal substitute for Sc [11,12,13,14,15]. Many scientific researchers have found that the grains can be refined and the corrosion resistance of the 7xxx aluminum alloy can be enhanced by adding a trace amount of Er into it [16,17]. The refining effect of Er on the alloy is mainly derived from the primary and secondary phases of Al_3_Er; however, Er also generates large insoluble Al_8_Cu_4_Er phases when combined with Cu in the alloy. Such phases serve as initial corrosion points, which degrade the corrosion resistance of the alloy [18,19,20,21,22]; however, the formation and the interaction of the Al_3_Er and Al_8_Cu_4_Er phases in the Er addition process has been rarely investigated.

Investigations in this study were performed with a self-made high-strength Al–Zn–Mg–Cu alloy with and without the addition of different amounts of Er. By adding variable amounts of the Er element we emphasize the study of the existing form of Er, the formation of Er-containing phases, and the internal relations between the alloy’s elements. Finally, the influence of phase evolution on the corrosion resistance of the alloy is described.

## 2. Materials and Methods

The alloy ingot used in this experiment was prepared by a traditional metal mold casting process. High purity Al (99.9 wt.%), Zn (99.9 wt.%), Mg (99.9 wt.%), and Al-50Cu (wt.%), Al-20Er (wt.%), Al-2Sc (wt.%), and Al-5Zr (wt.%) master alloys were added to prepare the alloys. The actual chemical compositions were detected by an XRF-1800 X-ray fluorescence spectrometer (Shimadzu, Kyoto, Japan), and are shown in Table 1 and denoted by #1, #2, #3, and #4, with Er content of 0, 0.10, 0.18, and 0.41 wt.%, respectively.

A well resistance furnace (SG-5-10) was used for smelting, and an iron mold and graphite crucible were also used in the process. During the smelting process, the stirring, refining, and pouring temperatures were kept in the range of 720 °C to 740 °C. In order to ensure the accuracy of the experiment, samples from the same position were selected for the next step. Afterwards, the as-cast ingots were treated with a two-stage solution (400 °C/4 h + 470 °C/48 h) and the water was left to cool.

Subsequently, the samples were tested for intergranular corrosion (IGC) according to the GB/T 7998-2005 standard. During tests, the samples were immersed in the corrosive medium of a 10 mL/L H_2_O_2_ and NaCl (57 g/L) solution for 6 h at 35 ± 2 °C. The sizes of the tested samples were 10 × 10 × 20 mm. After the IGC tests, the tested samples were mounted for protection and 5 mm was ground off along the length of each sample. The maximum depths of the IGC tests were analyzed by optical metallography (OM, LEICA DMi8).

Open-circuit potential (OCP) tests were performed on a Gamry reference 600 electrochemical workstation for 540 s to establish an approximate steady state. Following this, potentiodynamic polarization tests were performed from a starting potential of −1.1 V_SCE_ to an end potential of −0.7 V_SCE_, with a scan rate of 1 mVs^−1^. The electrochemical tests were performed in a 3.5%NaCl solution at room temperature. The samples used in the test were at a solid solution state and were ground with SiC sandpaper and flannel polished before testing. The exposed area for the working electrode was 1 cm^2^, and the electrolyte was 300 mL. Three tests were performed for each sample condition to investigate the accuracy of the results.

An X-ray diffractometer (XRD, D8 Advance, Billerica, MA, USA) with Cu-*K*α radiation was used for phase analysis of the as-cast and solid solution alloys. The microstructures were analyzed using electron probe microscopy (EPMA, JXA-8530F PLUS, Tokyo, Japan), operating at 15 kV. The second-phase compositions and micro-element distributions were analyzed by wavelength dispersive spectrum (WDS). Image-Pro Plus software was used to analyze the volume fraction of the second phase.

## 3. Results and Discussion

### 3.1. As-Cast and Solid Solution-State Microstructural Characteristics of Alloy

**Figure 1:**
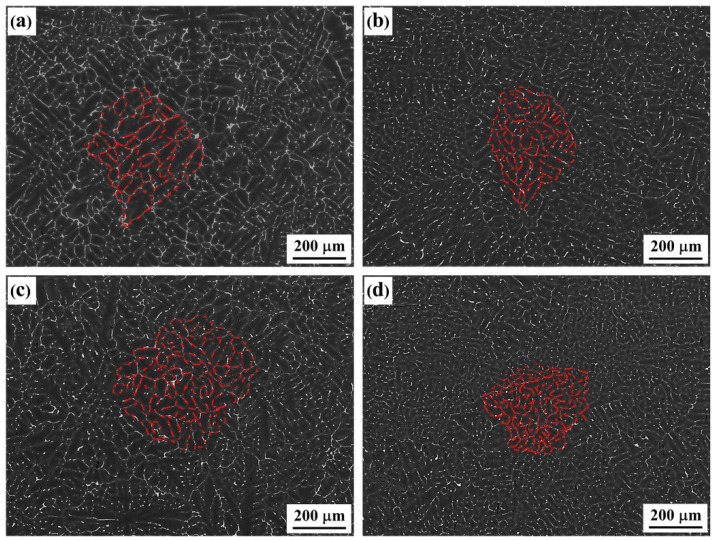
Backscattered electron (BSE) image of the as-cast alloys: (**a**) Er-free; (**b**) 0.1% Er; (**c**) 0.2% Er; (**d**) 0.4% Er alloys.

The backscattered electron images (BSE) of as-cast alloys with differing Er contents are shown in Figure 1. The figure indicates that the dendritic structure played a dominant role among the alloys, with four types of composition. As the Er content was increased, the refining–coarsening–refining evolutionary features were presented (as highlighted in red). In the #2 and #4 alloys containing 0.1% and 0.4% of added Er element, the overall structure was refined, the dendrite spacing was reduced, and the dendritic segregation was relieved, which is consistent with reports by other scholars [19]. Furthermore, the continuous grain boundary phases in the original #1 alloy gradually disappeared. In the #3 alloy containing 0.2% of added Er, the overall structural characteristics were basically consistent with those in the #1 alloy; however, the continuity of second phases was weakened to a certain extent.

**Figure 2:**
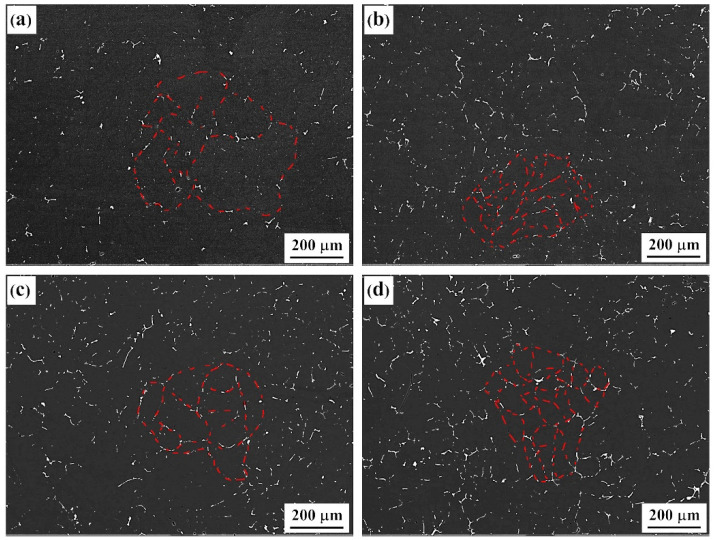
Backscattered electron (BSE) image of the solid solution alloys: (**a**) Er-free; (**b**) 0.1% Er; (**c**) 0.2% Er; (**d**) 0.4% Er alloys.

The BSE images of solid-solution-state alloys containing differing Er contents are presented in Figure 2. After the solid-solution treatment of the alloy, a large number of second phases at the original grain boundary disappeared in the solid solution, and undissolved second phases were left at the grain boundary to different degrees. The second-phase distribution shows that the alloy grains could be effectively refined by adding the Er element; this was most evident in the #4 alloy, which contained 0.4% Er. We calculated the proportions of the second phase using the Image-Pro analysis software; these were 0.56%, 0.91%, 0.97%, and 1.39%, respectively. By combining Figure 1 and Figure 2, we find that although the quantity of second phases at the grain boundary was reduced greatly after the Er addition, some insoluble phases were also generated. The quantity of residual second phases in the #2 alloy containing 0.1% Er was obviously increased. Moreover, the quantity of residual second phases changed minimally under different Er contents, and only in the #4 alloy containing 0.4% Er were the second phases obviously coarsened, as shown in Figure 2d.

### 3.2. Types of Evolution Characteristics of Second Phases at the Grain Boundary

**Figure 3:**
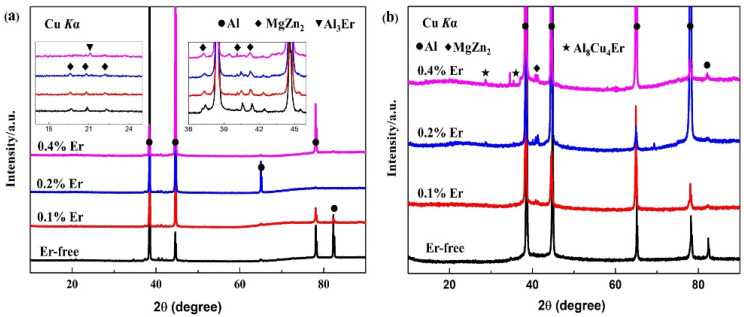
XRD patterns of studied alloys of (**a**) as-cast state; (**b**) solution state.

According to the as-cast and solid solution-state XRD diffraction results in Figure 3, the as-cast alloy with each composition mainly consisted of an α-Al phase and an η (MgZn_2_) phase, and the Al_3_Er phase appeared only when the Er content reached 0.4%. After the solid-solution treatment, the diffraction peak of the η (MgZn_2_) phase in the #1 alloy disappeared, indicating that the grain boundary phases were basically dissolved in α-Al. With the addition of Er and the increase in its content, the η (MgZn_2_) phase appeared simultaneously with the Al_8_Cu_4_Er phase and presented a gradually increasing tendency. In the meantime, the Al_3_Er phase discovered in the #4 as-cast alloy could hardly be detected by the XRD. Being an insoluble phase, the Al_3_Er phase formed a dependence relation with the Al_8_Cu_4_Er phase in the solid-solution treatment.

**Figure 4:**
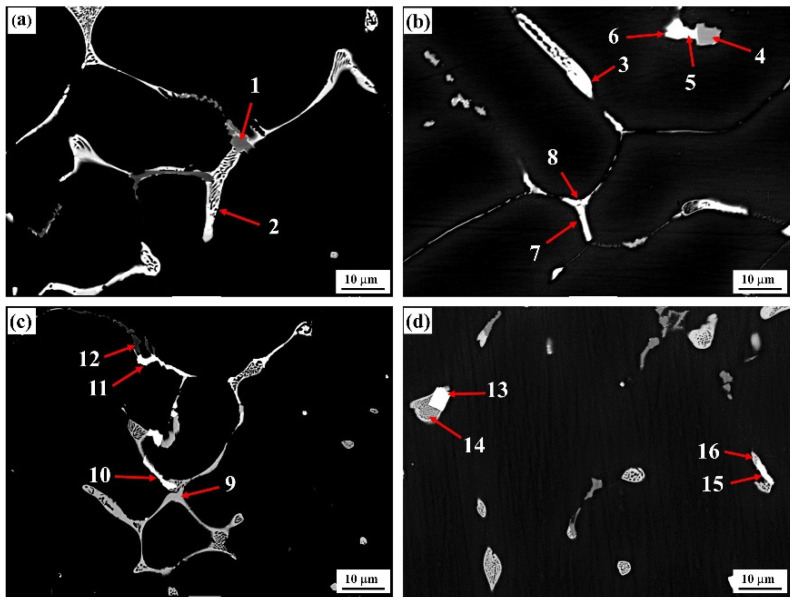
Backscattered electron (BSE) image of the as-cast alloys: (**a**) Er-free; (**b**) 0.1% Er; (**c**) 0.2% Er; (**d**) 0.4% Er alloys.

The typical grain boundary phase morphologies in as-cast alloys with differing Er contents and the corresponding alloy compositions are presented in Figure 4 and Table 2. Figure 4a shows that the bright white second phase continuously distributed at the grain boundary (position 2) in the #1 alloy was of a reticular structure. Compared with Al, Zn, Mg, and Cu, this phase should be a T (AlZnMgCu) nonequilibrium eutectic phase [23]. Meanwhile, we know from the XRD graphs that only the diffraction peak of MgZn_2_ existed in the as-cast alloy instead of the diffraction peaks of α-Al and Al_3_Er, indicating that this quaternary phase had an MgZn_2_ structure, and that the partial Zn atoms in the atomic lattice were substituted by Al and Cu atoms to form the Mg (Zn, Cu, Al)_2_ solid solution. In addition, a small quantity of gray Al_3_Fe phase intermingled between T-phases also existed in the alloy. In this phase, a certain amount of Cu element was dissolved, as indicated at position 1 in Figure 4a.

The addition of the Er element evidently changed the composition and morphology of the second phases at the grain boundary in the alloy. When 0.1% Er was added, the original reticular phase in the #2 alloy gradually transformed into skeleton and rodlike structures. In this condition, the Cu content was significantly increased, but the Zn and Mg contents were reduced. The type of eutectic phase was gradually transformed from a T-phase into an Al–Cu–Er phase, as denoted at position 3. Such a phase transition process was verified by the compositional variation at two groups of positions: 5 and 6 and 7 and 8, which were very approximate in morphology and position.

The quantity of T-phases (gray contrast, Position 9) was obviously increased in the #3 alloy, which was consistent with the structural evolution (increased in second phases), as described in Figure 1c. At the time, a small quantity of the Al_3_Er phase appeared simultaneously. Its morphological structure was basically identical to the Al–Cu–Er phase at position 10, such that it could hardly be differentiated from the microstructural graph. Moreover, this phase was not discovered in the XRD graph of the as-cast alloy in Figure 3a either, indicating that this phase was only formed in an insufficient quantity. The as-cast second phases were significantly reduced in the #4 alloy, and the residual second phases were still dominated by the Al–Cu–Er phase. Further, the square Al_3_Er phase differs obviously from the Al–Cu–Er phase that was discovered in the alloy, which was identical with the XRD result. Nevertheless, this phase still forms a dependence relation with the Al–Cu–Er phase.

**Figure 5:**
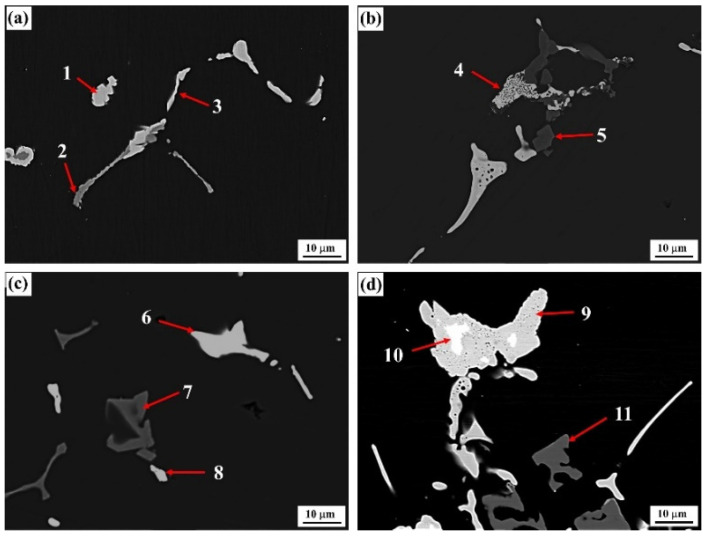
Backscattered electron (BSE) image of the solid solution alloys: (**a**) Er-free; (**b**) 0.1% Er; (**c**) 0.2% Er; (**d**) 0.4% Er alloys.

The typical grain boundary phase morphologies of solid-solution-state alloys with differing Er contents and their corresponding alloy compositions are shown in Figure 5 and Table 3. After the solid-solution treatment, the original T-phases in the #1 alloy disappeared in the solid-solution treatment, the small quantity of residual phases were mainly Fe-containing second phases, and the skeletonlike phase was mainly an Al–Cu–Fe phase, which might be attributed to the gradual aggregation of Cu and Fe by partial Cu-containing phases adjacent to the Al–Fe phase in the solid-solution treatment. In the #2 and #3 alloys containing 0.1% and 0.2% Er, only one new phase was left at the grain boundary with the exception of Al–Fe phase, that is, an Al_8_Cu_4_Er phase, which was transited from an as-cast Al–Cu–Er phase; this result was identical to the XRD result in Figure 3b. In the #2 alloy containing a low Er content, this phase also presented reticular structural features; however, in the #3 alloy containing a higher Er content, this phase was closer to the massive phase. The Al_3_Er phase discovered under the as-cast state of the #3 alloy could hardly be found at the time. With a further increase in the Er content, the residual phases at the grain boundary of the #4 alloy were obviously coarsened; meanwhile, the Al_3_Er phase wrapped in the Al_8_Cu_4_Er phase was also found.

**Figure 6:**
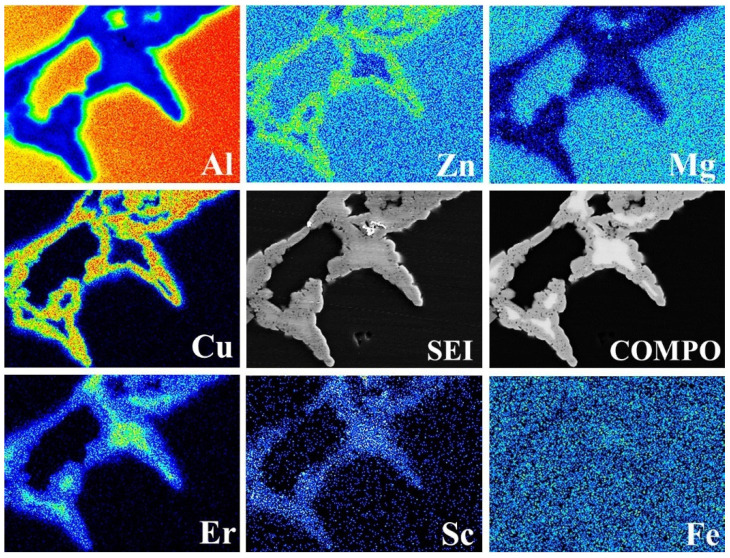
Mapping of the 0.4% Er alloy.

The formation and growth of the Al_3_Er phase were closely related to the Al_8_Cu_4_Er phase, which presented an obvious growth-dependent relation. To study the in-depth interaction between the two phases, a micro-area chemical analysis was performed for typical positions in the #4 solid solution-state alloy. The results are shown in Figure 6. Er was mainly distributed in the central phase-area with bright contrast, and the contents of the other alloy elements in this area were very low, indicating that this phase was an Al_3_Er phase. The surrounding area with relatively dark contrast was a mixed area of Zn, Mg, Cu, and Er, with the component being a typical Al_8_Cu_4_Er phase.

These results indicate that the Al_8_Cu_4_Er and Al_3_Er phases have a symbiotic competitive growth relation. With a high amount of the Er element (0.2%), the conditions for the initial formation of Al_3_Er were met; a large amount of Er was then consumed and it did not interact with Zn, Mg, or Cu, thereby inhibiting the formation of the Al–Cu–Er phase. As a result, the T-phases in the #3 as-cast alloy were obviously increased compared with those in the #2 alloy. Meanwhile, the refining effect of the Al–Cu–Er phase on the grains was weakened so that the dendrites in the as-cast #3 alloy were coarsened in comparison with those in the #2 alloy. As the Er content was further increased, a large quantity of the Al_3_Er phase was generated, thereby exerting a significant refining effect on the grains. As the formation and growth of the Al_3_Er phase under the as-cast state depended on the Al–Cu–Er phase, the formation of this phase significantly repressed the formation and growth of the Al_3_Er phase during the solid-solution treatment that transformed Al–Cu–Er into a steady Al_8_Cu_4_Er phase, and even decomposed and absorbed the original as-cast Al_3_Er phase in the #3 alloy. Only in the core of the coarsened Al_8_Cu_4_Er phase in the #4 alloy was a small quantity of incompletely decomposed Al_3_Er phase found; thus, the two presented obvious competitive growth characteristics.

### 3.3. Corrosion Resistance

**Figure 7:**
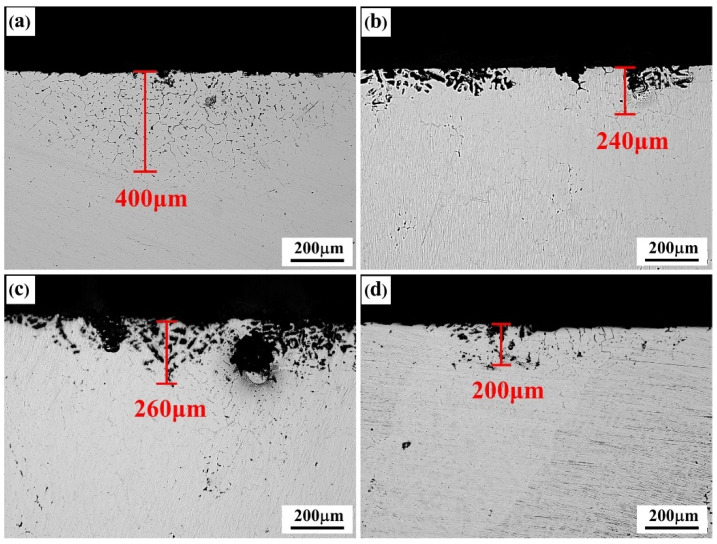
IGC morphologies of the as-cast alloys: (**a**) Er-free; (**b**) 0.1% Er; (**c**) 0.2% Er; (**d**) 0.4% Er alloys.

**Figure 8:**
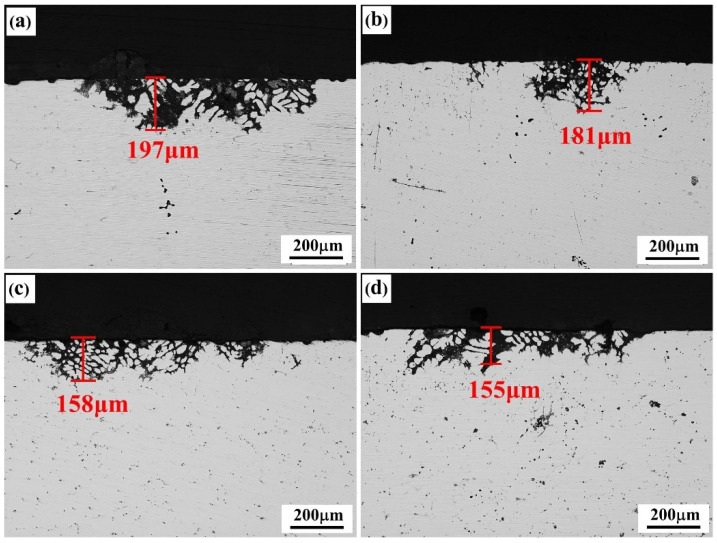
IGC morphologies of the solid solution alloys: (**a**) Er-free; (**b**) 0.1% Er; (**c**) 0.2% Er; (**d**) 0.4% Er alloys.

The metallographs of intergranular corrosion sections of as-cast and solid-solution-state alloys with differing Er contents are shown in Figure 7 and Figure 8, respectively. The as-cast alloys with differing Er contents experienced the intergranular corrosion to varying degrees, among which the #1 alloy containing no Er presented typical intergranular corrosion characteristics, with the corrosion depth reaching 400 μm. The intergranular corrosion resistance of the alloy was significantly enhanced by adding the Er element, and pitting corrosion played a dominant role. With the increase in Er content, the corrosion pit depth of the alloy showed a declining–increasing–declining tendency. The intergranular corrosion depth was approximately 200 μm in both the #2 and #4 alloys containing 0.1% and 0.4% Er, respectively, and 260 μm in the #3 alloy containing 0.2% Er.

The corrosion resistance of the alloys was strengthened to various degrees after the solid-solution treatment. The pitting corrosion was the main corrosion feature, and no obvious intergranular corrosion occurred, indicating that this type of alloy had high intergranular corrosion resistance; however, the pitting corrosion of the alloy was significantly influenced by the second phases at the grain boundary. The quantity of insoluble second phases in the alloy increased with the addition of Er content, but its pitting corrosion resistance was strengthened to various degrees. The degree of corrosion of the #1 alloy containing no Er content was the most serious, with the corrosion depth reaching 197 μm. The degree of corrosion of the #2 alloy containing 0.1% Er was slightly relieved, and the corrosion depth was reduced to 181 μm. The pitting corrosion resistance of the #3 alloy containing 0.2% Er was basically equivalent to that of the #4 alloy containing 0.4% Er content, and their corrosion depths were 158 μm and 155 μm, respectively.

The precipitated phases continuously distributed at the grain boundary easily formed corrosion channels in the corrosive medium, which facilitated the intergranular corrosion in the alloy and accelerated the alloy corrosion [24,25]. The microstructure of the as-cast alloy shows that continuous grain boundary phases existed in the alloy containing no Er. After the Er element was added, both the grains and second phases at the grain boundary in the as-cast alloy were refined, and no continuously distributed precipitated phases at the grain boundary were formed; thus, the intergranular corrosion resistance of the alloy was enhanced. After the solid-solution treatment, the corrosion resistance of the alloy was markedly affected by the type, morphology, size, and distribution of the residual second phase.

**Figure 9:**
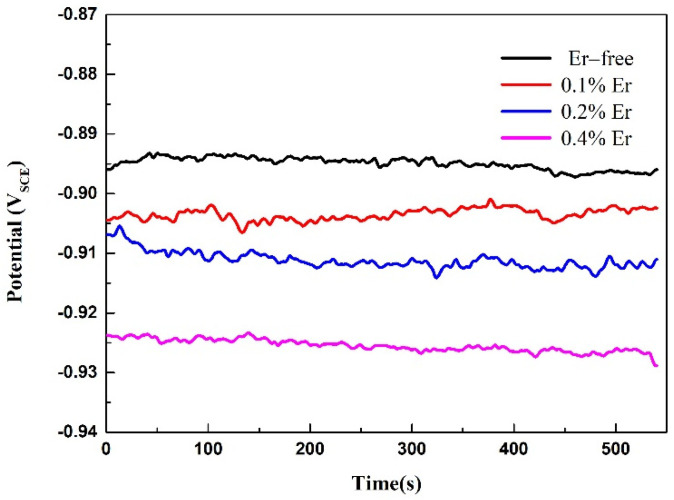
OCP test results of the solid−solution alloys.

**Figure 10:**
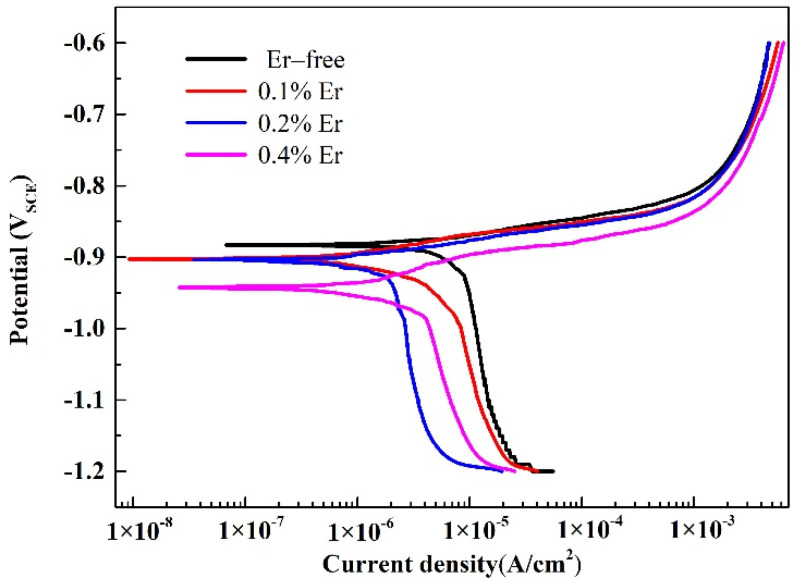
Polarization curves of the solid−solution alloys.

To further study the effect of various Er contents on the corrosion resistance of solid-solution-state alloys, OCP and potentiodynamic polarization tests were performed, and the results are shown in Figure 9 and Figure 10. The corresponding corrosion potential (*E*_corr_) and corrosion current density (*i*_corr_) of the alloy could be obtained by fitting the polarization curves, as reported in Table 4. As shown in Figure 9, with the increase in Er content, the OCP decreased gradually. The table shows that with no Er content, the alloy reached the maximum corrosion potential and corrosion current density. When the Er content was increased to 0.1%, the corrosion potential declined, and the corrosion current was also reduced; therefore, the corrosion resistance of the alloy was enhanced. Compared with the alloy containing 0.1% Er, the alloy containing 0.2% Er showed no obvious changes in the corrosion potential or corrosion current density. In the alloy containing 0.4% Er, both the corrosion potential and corrosion current density were substantially reduced.

Corrosion potential is a thermodynamic parameter that describes the corrosion tendency of alloys; the more negative its value is, the higher the corrosion tendency [26,27]. Corrosion current density is a kinetic parameter that describes the corrosion of alloys; the greater its value is, the faster the alloys are corroded. In the #1 alloy containing no Er, the residual grain boundary phases were dominated by the Al–Cu–Fe and Al–Fe phases, both of which could serve as the cathode to constitute a corrosion cell with anodic α-Al [28]; consequently, the surrounding grains were the first to undergo corrosion, and the pitting corrosion resistance of the #1 alloy was the poorest.

The corrosion potential of the #2–#4 alloys was reduced because Al_8_Cu_4_Er and Al_3_Er phases were formed due to the addition of Er, whose electrode potentials were both lower than that of the matrix. The appearance of the two phases enhanced the potential difference between the grain boundary and the matrix, and they could be the anode in the corrosion process to change the grain boundary corrosion mode [29]. The actual corrosion rates of the alloys with three differing Er contents were significantly reduced; thus, the corrosion resistance of the alloy was obviously improved by the Al_8_Cu_4_Er and Al_3_Er phases; however, this improvement was restricted by the phase morphology and distribution in the alloy. In the #2 alloy, the skeleton or reticular Al_8_Cu_4_Er phase was formed, which led to the poor corrosion resistance of the alloy. The pitting corrosion resistance was basically equivalent to that of the matrix, and the short rodlike or irregular square Al_8_Cu_4_Er and Al_3_Er phases could effectively reduce the pitting corrosion tendency of the alloy. When the Er content was increased from 0.2% to 0.4%, the alloy grains were obviously refined; however, the residual insoluble phases were coarsened such that the intergranular corrosion resistance was no longer improved.

Both the Al_8_Cu_4_Er and Al_3_Er phases exerted a refining effect on the alloy, and the key to enhancing the corrosion resistance of the alloy was to obtain refined and dispersed Er-containing rare earth phases. The competitive growth mechanism of the two phases of the formation of the Al_8_Cu_4_Er phase was closely related to the enrichment and redistribution of the Cu element. As shown in Figure 4, the T-phases were transformed into Al_8_Cu_4_Er phases at positions 13 and 14. Obviously, compared with the Al_3_Er phase, the Al_8_Cu_4_Er phase could easily interact with the Cu element in the formation and growth process, and was then coarsened, thereby inhibiting the formation of the Al_3_Er phase; thus, to exert the improving effect of the Er element on the corrosion resistance of the Al–Zn–Mg–Cu aluminum alloy to the greatest extent, we have to further study the matching design of Cu and Er as well as the growth mechanism of the Al_8_Cu_4_Er phase.

## 4. Conclusions

(1) As the Er content (0–0.4%) increased in the Al–Zn–Mg–Cu–Sc–Zr alloy, the types of second phases formed by Er control the microstructure; the dendrite arms and grains size were first refined, then coarsened and refined again.
(2) The interaction between Cu and Er can form the ternary Al_8_Cu_4_Er phase in the Al–Zn–Mg–Cu–Sc–Zr alloy; however, when the Er content increased, the primary Al_3_Er phase was formed in the center of the Al_8_Cu_4_Er phase, which showed an interactive and competitive growth relation with the Al_8_Cu_4_Er phase.
(3) The Al_8_Cu_4_Er and Al_3_Er phases enhanced the corrosion resistance of the alloy by changing the potential difference between the grain boundary phases and the matrix; however, higher Er content lead to the coarsening of the Er-containing phase, which inhibited the further improvement of the corrosion performance of the alloy.
(4) To enhance the corrosion resistance of the alloy, it is important to control the interaction between the Al_8_Cu_4_Er phase and Cu during its formation and growth process. In the future, by studying the effect of Cu enrichment and redistribution on the Al_8_Cu_4_Er phase, a finely dispersed Er-containing rare earth phase can be obtained, thereby improving the corrosion performance of the alloy.

## Figures and Tables

**Table 1:** Chemical composition of the experimental alloy (wt.%).

Alloys	Zn	Mg	Cu	Sc	Zr	Er	Fe	Al
#1	8.80	1.73	0.99	0.13	0.11	-	0.11	Bal.
#2	8.92	1.77	1.03	0.13	0.09	0.10	0.13	Bal.
#3	8.77	1.79	1.00	0.08	0.13	0.18	0.12	Bal.
#4	9.01	1.87	1.02	0.08	0.10	0.41	0.16	Bal.

**Table 2:** Chemical compositions (wt.%) of points marked in Figure 4.

Alloys	Points	Phase Type	Al	Zn	Mg	Cu	Er	Fe
a	1	Al–Fe	64.47	1.99	0.65	5.29	-	27.57
	2	T	41.4	29.92	20.83	7.71	-	-
b	3	Al–Cu–Er	50.51	11.1	4.51	27.19	4.32	1.72
	4	Al–Fe	67.22	1.77	0.75	3.37	-	26.77
	5	T	30.41	29.43	28.03	10.62	0.16	1.31
	6	Al–Cu–Er	47.97	11.33	6.72	29.03	3.02	1.52
	7	T	58.04	19.82	17.75	4.36	-	-
	8	Al–Cu–Er	68.65	7.34	2.78	17.34	2.41	1.05
c	9	T	26.60	33.36	31.86	7.91	0.01	0.01
	10	Al–Cu–Er	48.18	9.92	2.23	32.54	4.78	1.84
	11	Al_3_Er	72.68	2.41	0.53	0.54	17.03	2.69
	12	Al–Fe	66.11	1.92	0.32	3.53	-	28.1
d	13	Al_3_Er	73.69	2.81	1.42	0.10	19.51	0.08
	14	T	52.13	21.41	20.50	5.52	0.35	0.08
	15	Al–Cu–Er	55.30	9.52	1.20	26.53	6.09	1.14
	16	T	48.61	25.80	20.31	4.84	0.30	0.10

**Table 3:** Chemical compositions (wt.%) of points marked in Figure 5.

Alloys	Points	Phase Type	Al	Zn	Mg	Cu	Er	Fe
a	1	Al–Cu–Fe	71.87	1.18	0.03	16.58	-	10.31
	2	Al–Fe	76.40	1.69	0.45	2.39	-	15.94
	3	Al–Cu–Fe	73.87	1.34	0.24	15.37	-	9.11
b	4	Al_8_Cu_4_Er	57.78	8.55	1.05	26.15	5.40	0.51
	5	Al–Fe	78.46	2.87	2.41	0.82	0.41	12.63
c	6	Al_8_Cu_4_Er	61.77	8.86	1.59	22.09	4.68	0.54
	7	Al–Fe	75.40	1.91	0.52	2.30	-	16.58
	8	Al_8_Cu_4_Er	59.37	8.94	1.84	24.02	4.75	0.61
d	9	Al_8_Cu_4_Er	53.24	11.37	0.08	26.44	8.05	0.11
	10	Al_3_Er	73.03	2.08	0.31	1.14	19.87	0.04
	11	Al–Fe	75.99	2.17	1.07	2.74	0.05	17.64

**Table 4:** Electrochemical parameters of alloys obtained from polarization tests.

Alloys	*E*_corr_ (V_SCE_)	*i*_corr_ (μA/cm^2^)
#1	−0.88	8.3
#2	−0.90	5.9
#3	−0.90	5.7
#4	−0.94	3.4

## Data Availability

The raw/processed data required to reproduce these findings cannot be shared at this time due to technical or time limitations.
